# Role of Diffusion Tensor Imaging in Prognostication and Treatment Monitoring in Niemann-Pick Disease Type C1

**DOI:** 10.3390/diseases4030029

**Published:** 2016-09-08

**Authors:** Meghann W. Lau, Ryan W. Lee, Robin Miyamoto, Eun Sol Jung, Nicole Yanjanin Farhat, Shoko Yoshida, Susumu Mori, Andrea Gropman, Eva H. Baker, Forbes D. Porter

**Affiliations:** 1 Research Department, Shriners Hospitals for Children, Honolulu, HI 96822, USA; meghann.lau@gmail.com (M.W.L.); rmiyamoto@shrinenet.org (R.M.); 2 John A. Burns School of Medicine, University of Hawaii, Honolulu, HI 96813, USA; 3 Department of Neurology, Shriners Hospitals for Children, Honolulu, HI 96822, USA; 4 Department of Pediatrics, John A. Burns School of Medicine, University of Hawaii, Honolulu, HI 96813, USA; 5 Department of Psychiatry, Kennedy Krieger Institute, Baltimore, MD 21205, USA; JungES@kennedykrieger.org; 6 *Eunice Kennedy Shriver* National Institute of Child Health and Human Development, National Institutes of Health, Bethesda, MD 20892, USA; nicole.farhat@nih.gov (N.Y.F.); fdporter@mail.nih.gov (F.D.P.); 7 Department of Radiology, School of Medicine, Johns Hopkins University, Baltimore, MD 21205, USA; shokoyoshida828@gmail.com (S.Y.); susumu@mri.jhu.edu (S.M.); 8 Division of Neurogenetics, Children’s National Medical Center, Washington, DC 20010, USA; agropman@childrensnational.org; 9 Department of Radiology and Imaging Sciences, National Institutes of Health, Bethesda, MD 20892, USA; bakere@cc.nih.gov

**Keywords:** Niemann-Pick, NPC, diffusion tensor imaging, DTI, cerebellum, motor function

## Abstract

Niemann-Pick Disease, type C1 (NPC1) is a rapidly progressive neurodegenerative disorder characterized by cholesterol sequestration within late endosomes and lysosomes, for which no reliable imaging marker exists for prognostication and management. Cerebellar volume deficits are found to correlate with disease severity and diffusion tensor imaging (DTI) of the corpus callosum and brainstem, which has shown that microstructural disorganization is associated with NPC1 severity. This study investigates the utility of cerebellar DTI in clinical severity assessment. We hypothesize that cerebellar volume, fractional anisotropy (FA) and mean diffusivity (MD) negatively correlate with NIH NPC neurological severity score (NNSS) and motor severity subscores. Magnetic resonance imaging (MRI) was obtained for thirty-nine NPC1 subjects, ages 1–21.9 years (mean = 11.1, SD = 6.1). Using an atlas-based automated approach, the cerebellum of each patient was measured for FA, MD and volume. Additionally, each patient was given an NNSS. Decreased cerebellar FA and volume, and elevated MD correlate with higher NNSS. The cognition subscore and motor subscores for eye movement, ambulation, speech, swallowing, and fine motor skills were also statistically significant. Microstructural disorganization negatively correlated with motor severity in subjects. Additionally, Miglustat therapy correlated with lower severity scores across ranges of FA, MD and volume in all regions except the inferior peduncle, where a paradoxical effect was observed at high FA values. These findings suggest that DTI is a promising prognostication tool.

## 1. Introduction

Niemann-Pick disease type C (NPC) is an autosomal recessive lysosomal storage disorder, characterized by a mutation in NPC1 or NPC2, resulting in an accumulation of intracellular unesterifified cholesterol and lipids [1]. The estimated prevalence of NPC is 1 in 100,000 European births, with Niemann-Pick disease type C1 (NPC1) accounting for 95% of cases, and Niemann-Pick disease type C2 (NPC2), 5% [2,3]. NPC1 arises from a mutation in NPC1, which encodes for a transmembrane protein incorporated into late endosomes [4]. When NPC1 is dysfunctional, lipids accumulate in the endosomal system of the cell, which precipitates a cascade of cellular events, leading to neuronal death. The clinical progression of NPC often begins with prolonged neonatal jaundice, followed by relatively normal early childhood development before the observable onset of ataxia, dysarthria, dementia, seizures, dystonia and neurocognitive deficits. These signs are commonly accompanied by hepatosplenomegaly and can occur at any age, but are most commonly present in children [5].

In early onset NPC, disease progression is rapid, and expanding our arsenal of severity markers is essential to improving care. Increased serum levels of galectin-3 (LGALS3), a pro-inflammatory molecule, and cathepsin D (CTSD), a lysosomal aspartic protease, as well as various cholesterol oxidation products have been found to be potential serum biomarkers in murine models that may aid in diagnosis and therapeutic monitoring in NPC1 patients [6,7]. Imaging markers are yet to be developed. Lee et al. (2014) found decreased volume and microstructural organization of the corpus callosum were associated with increased NIH NPC severity scores (NNSS) [8]. Cerebellar volume and fractional anisotropy (FA) have been shown to be significantly decreased in adult NPC patients [9,10]. However, there have been no publications to date documenting the in vivo cerebellar microstructural abnormalities and their clinical consequences in NPC patients using DTI [11,12]. This is the first study that aims to examine the relationship between cerebellar DTI measures and NPC1 disease severity in a pediatric population. A secondary analysis then examined the effects of miglustat status on these DTI measures. The DTI measures in this study include FA, a measure of diffusion directionality, and mean diffusivity (MD), and a measure of diffusion magnitude. Both are measures of brain microstructural abnormality that are not identified by routine MRI scans. Lower FA and higher MD represent microstructural disorganization in the region examined and are associated with impaired neuronal conduction. We hypothesize that decreased FA and increased MD is associated with increased NNSS and particularly, increased motor severity subscores.

## 2. Materials and Methods

### 2.1. Study Sample

The *Eunice Kennedy Shriver* National Institute of Child Health and Human Development, Institutional Review Board (Bethesda, MD, USA) reviewed and approved this study. Parents or legal guardians voluntarily enrolled their children, provided written informed consent, and assent was obtained when possible. Thirty-nine pediatric subjects diagnosed with NPC1 following clinical examination and biochemical or genetic testing comprised this study’s sample.

### 2.2. Image Acquisition and Analysis

Between August 2006 and January 2012, one MRI scan was performed on each patient, each sedated with propofol for the duration of the scan. A Philips Achieva 3.0T magnetic resonance scanner (Philips, Amsterdam, Netherlands) was used to obtain axial magnetization-prepared rapid-gradient echo (MPRAGE), T2-weighted imaging (T2WI), and DTI sequences, without gaps. Acquisition parameters used for T2WI and DTI can be found in Table 1. Diffusion weighting was performed along 16 axes with a b value of 800 s/mm^2^. The automated atlas-based segmentation of the cerebellum was based on the T2-weighted images, which uses a reference map in place of explicit anatomic landmarks to generate sections.

A scanner upgrade occurred in 2010, and the resulting changes in MPRGE scan parameters can be found in Table 2.

Hardware and software updates that occurred while the scans were performed did not affect fractional anisotropy values, as the same b value was used throughout. Volume measurements were obtained by counting the number of voxels in the original image that mapped into the atlas-defined segment.

As the shortest TE is desired when using DTI, we minimized the image matrix and used a relatively small b value in our study to shorten TE as much as possible. The shortest TE, without compromising diffusion weighting, is desired, so a compromise was achieved between enough diffusion weighting and signal to noise ratio (SNR). Additionally, the use of a Philips scanner, which uses the single spin-echo sequence rather than the dual spin-echo sequence, which is an option used by Siemens and GE scanners, further contributed to a shorter TE. With a shorter TE, the signal:noise ratio is expected to be higher due to T2-relaxation decay. DTI is designed to achieve the shortest TE possible, as determined by gradient strength, gradient hardware performance, used pulse sequence, image matrix size, bandwidth, the skew rate, and parallel imaging factor. A comprehensive analysis of the signal:noise ratio and bias using similar imaging protocol has been published [13].

All DTI data sets were processed offline using DTIStudio software. Using a 12-mode affine transformation of automated image registration, the raw diffusion-weighted images were coregistered to a b = 0 image [14]. The six independent elements of the diffusion tensor (Dxx, Dyy, Dzz, Dxy, Dxz, Dyz), including FA and MD were then calculated. The FA and MD values were determined as an average of the region of interest (e.g. cerebellum). Large deformation diffeomorphic metric mapping (LDDMM) was performed to correct distortion caused by b0 susceptibility in accordance with a previous publication [15], using T2WI as the target and the b = 0 image as the DTI data. After skull stripping, the images were normalized to the JHU-DTI MNI “Eve” template with a nine-parameter affine transformation of automated image registration by using b0 images after distortion correction. A nonlinear transformation, accomplished by dual-contrast LDDMM, was applied [16,17] using FA and Trace images as two channel inputs. The nonlinear image transformation and the atlas-based parcellation were performed using DiffeoMap and RoiEditor [14,18,19,20]. Because these procedures are reciprocal, the inverse-transformed brain parcellation map was superimposed onto the original diffusion tensor images and led to parcellation of the brain into 130 anatomic structures [18,19,20]. After the quantitative values were obtained, the cerebrospinal fluid spaces were excluded by a mean diffusivity threshold set at 0.0020 mm^2^/s. Data were excluded based on motion degradation and poor image quality, as assessed by a neuroradiologist (EB) and neurologist (SY) with specialty in DTI.

A parcellation map was thus created to measure cerebellar FA and volume for each patient. The map enabled segmentation of the cerebellum and its associated peduncles into ten regions, defining right and left halves of each structure relative to the midline: left cerebellum, right cerebellum, left superior cerebellar peduncle, right superior cerebellar peduncle, left middle cerebellar peduncle, right middle cerebellar peduncle, left inferior cerebellar peduncle, right inferior cerebellar peduncle, left cerebellar white matter and right cerebellar white matter. FA and volume were determined for each region.

### 2.3. Severity Score, DTI and Volume Measurements

A neurological severity scoring system for NPC based on clinical evaluation was developed to aid monitoring the course of the disease. The system assigns an NNSS to each patient, derived from the sum of 17 subcategory severity scores (nine major and eight minor domains) in the following areas: eye movement, ambulation, speech, swallowing, motor, cognition, hearing, memory, seizures, cataplexy, narcolepsy, behavior, psychology, hyperreflexia, incontinence, respiratory, and auditory brain stem response. A patient’s score increases with disease severity, with a maximum score of 61 points, and the score is independent of age at onset of NPC, making it applicable to both children and adolescents [21]. In our study, NNSS, as well as all subscores, age at first symptoms, and duration of symptoms were analyzed in conjunction with DTI and volume.

### 2.4. Statistical Analyses

Spearman correlation assessed the associations between cerebellar FA, volume, and MD with NNSS. Raw DTI data were converted to population-averaged z-scores (standardized variables), which were calculated using an age-matched normal group as controls:

z = (x − μ)/σ
(1)

where μ was the mean of the control group and σ was the standard deviation of the control group. For the control group, the existing clinical database of pediatric MRIs with no remarkable findings was used. The clinical history of each patient was carefully evaluated by a neuropediatrician, and patients with apparent abnormal neurological findings, developmental abnormalities, or preterm birth history were excluded. Images were acquired using a 3T Siemens scanner. DTI: A single-shot echo planar imaging (EPI) with parallel acquisition; axial orientation; Imaging matrix, 96 × 96; FOV 240 × 240; thickness 2.5 mm; TE 84 ms; TR 7700 ms; diffusion-weighting along 21 axes with b = 1000 s/mm^2^; repeated twice to enhance the SNR. T2 FSE: whole brain; axial orientation; imaging matrix, 320 × 288; FOV 189 × 210 mm; thickness 3.5 mm; TE = 104 ms, TR = 4280 ms.

There is limited data on the effects of age and cerebellar development with respect to DTI changes. Z-scores were applied to correct for variability in age among imaging outcome measures. In addition, we performed a separate multiple regression analysis on the raw DTI data, and compared the results of the two statistical methods. For multiple regression analysis; NNSS and subscores (dependent variable) were each regressed on cerebellar FA, volume and MD and age at scan (independent variables and covariate).

Analysis of covariance (ANCOVA; general linear modeling (SAS/STAT^®^, 2011)) assessed the associations between miglustat therapy and NNSS and subscores, with cerebellar volume, FA, and MD as respective covariates. Each ANCOVA model tested the assumption (null hypothesis) that the relationships of NNSS and subscores with cerebellar volume, FA, or MD were statistically similar for, or didn’t differ among, those receiving vs. not receiving miglustat therapy.

A Wilcoxon-Mann-Whitney test was also performed to assess for different volume, FA and MD findings in the cerebellum according to a subject’s miglustat status (on vs. off). Two-sided *p*-value < 0.05 was considered to be statistically significant.

## 3. Results

### 3.1. Sample Demographics

This study’s sample was comprised of 35 pediatric and four young adult subjects diagnosed with NPC1 by clinical examination and cellular or genetic testing. There were 20 females and 19 males ranging in age between 1 and 21.9 years (mean 11.1, SD 6.1) (Table 3). The ethnic distribution was Caucasian (34, 87%), Hispanic (4, 10%), and Hispanic/American Indian (1, 2.6%). NNSS ranged from 1 to 46 points (mean 16.2, SD 11.5). The age at first neurological symptoms ranged from birth to 13 years (mean 2.7, SD 3.6). The duration of neurological symptoms ranged from 1.1 to 16.5 years at the time of scan (mean 7, SD 4.1). Common presenting symptoms included splenomegaly, jaundice, and hepatosplenomegaly. Of the twenty-one subjects, 54% were receiving off-label oral miglustat. Additional demographic information is provided in Table 3.

### 3.2. Cerebellar Fractional Anisotropy (FA)

The cerebellar FA of the right/left inferior and right/left superior peduncles, right white matter, and right/left/whole cerebellum were negatively correlated with NNSS (Table 4). Various cerebellar FA were negatively correlated with subscores for eye movement, ambulation, speech, swallowing, fine motor skills, cognition, seizures, psychology, respiratory, age at first neurological symptoms and duration of neurological symptoms (Supplementary Table S1). For example, FA of the right/left inferior and right superior peduncles, and right/left/whole cerebellum were also negatively correlated with eye movement subscores (Supplementary Table S1). There were no statistically significant correlations found between cerebellar FA and hearing, memory, cataplexy, narcolepsy, behavior, hyperreflexia, incontinence, and auditory brain stem response scores.

Multiple regression models of NNSS with the FA of the right inferior peduncle and right/left white matter, respectively, along with age at scan (covariate) were statistically significant (Table 5). Also, in models of eye movement (*f* = 5.74, *p* < 0.01, R^2^ = 0.24), ambulation (*f* = 5.79, *p* < 0.01, R^2^ = 0.24), and swallowing (*f* = 10.07, *p* < 0.001, R^2^ = 0.36) subscores with the right inferior peduncle and age at scan (covariate); of ambulation (*f* = 7.46, *p* < 0.01, R^2^ = 0.29), speech (*f* = 4.27, *p* < 0.05, R^2^ = 0.19), and swallowing (*f* = 10.57, *p* < 0.001, R^2^ = 0.37) subscores with the right white matter and age at scan covariate; and of ambulation (*f* = 8.56, *p* < 0.001, R^2^ = 0.32), speech (*f* = 6.35, *p* < 0.01, R^2^ = 0.26), swallowing (*f* = 10.11, *p* < 0.001, R^2^ = 0.36) subscores with the left white matter and age at scan covariate; were statistically significant. There were also statistically significant within-model associations between the eye movement (*f* = 6.26, *p* < 0.01, R^2^ = 0.26) subscores with the FA measure of the right white matter and age at scan covariate (negative parameter estimates) and between fine motor skills (*f* = 4.67, *p* < 0.05, R^2^ = 0.21) and memory (*f* = 3.30, *p* < 0.05, R^2^ = 0.15) subscores with the FA measure of the left white matter and age at scan covariate (positive parameter estimates) (Supplementary Table S2).

Wilcoxon-Mann-Whitney test results indicated no statistically significant difference between on and off miglustat groups in FA. ANCOVA models of NNSS in patients on miglustat, with covariate FA of the R/L inferior and L superior peduncles, were statistically significant (Table 6).

**Figure 1:**
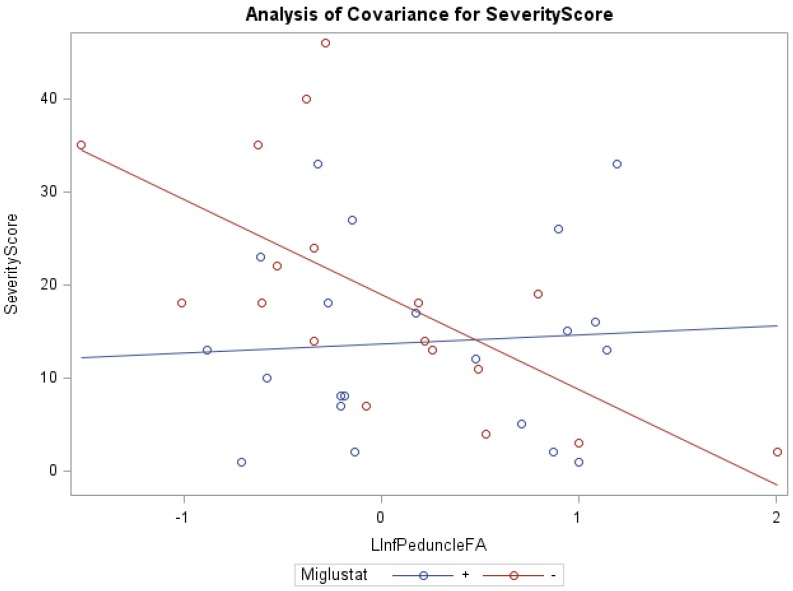
ANCOVA of NNSS, miglustat therapy, and FA of the left inferior peduncle.

In addition, ANCOVA of swallowing scores and miglustat therapy, with the covariates FA of the left inferior peduncle (*f* = 7.71, *p* < 0.001, R^2^ = 0.40) and right middle peduncle (*f* = 4.67, *p* < 0.05, R^2^ = 0.21) were statistically significant. Groups receiving versus groups not receiving miglustat therapy were found to have significantly lower swallowing subscores, with the FA of the left superior peduncle (0.82 vs. 1.87) and left middle peduncle (0.81 vs. 1.86), as respective covariates. The groups receiving miglustat therapy had significantly lower duration of neurological symptom subscores, with the FA of the left white matter (5.57 vs. 9.07), as the covariate. The statistically significant independent effect of miglustat therapy on psychology scores, and of FA for eye movement, ambulation, speech, cognition, age at first neurological symptoms, and duration of neurological symptom were also noted (Supplementary Table S3). There were statistically significant interaction effects between miglustat therapy with the covariates FA of the right/left (Figure 1) inferior peduncles for NNSS, swallowing, fine motor, cognition, psychology, and duration of neurological symptom (Supplementary Table S3).

### 3.3. Cerebellar Volume

The cerebellar volume of the right inferior and right/left superior peduncles were negatively correlated with NNSS (Table 4). Other cerebellar volumes were negatively correlated with eye movement, ambulation, speech, swallowing, fine motor skills, cognition, seizures, and hyperreflexia (Supplementary Table S1). Positive correlations were found between the volume of the right/left/whole cerebellum with ambulation and auditory brain stem response. There were no statistically significant correlations found between cerebellar volume and hearing, memory, cataplexy, narcolepsy, behavior, psychology, hyperreflexia, incontinence, respiratory, and duration of neurological symptom.

Multiple regression models of NNSS with the volume for the right/left inferior, right/left superior and right/left middle peduncles, and right/left white matter and age at scan (covariate) were statistically significant (Table 5). Statistically significant models of eye movement, ambulation, swallowing, fine motor skills, and cognition with the volume for the right/left superior peduncles and right/left white matter and age at scan (covariate) were found (Supplementary Table S2). Cerebellar volume of the right/left inferior peduncles were statistically significant (minimal) parameters, whereas age at scan was a statistically significant (>minimal) parameter, related to age at first neurological symptoms (positive direction) and duration of neurological symptoms (negative direction).

Wilcoxon-Mann-Whitney test results indicated no statistically significant differences in volume of various cerebellar regions between those on versus those off miglustat therapy.

**Figure 2:**
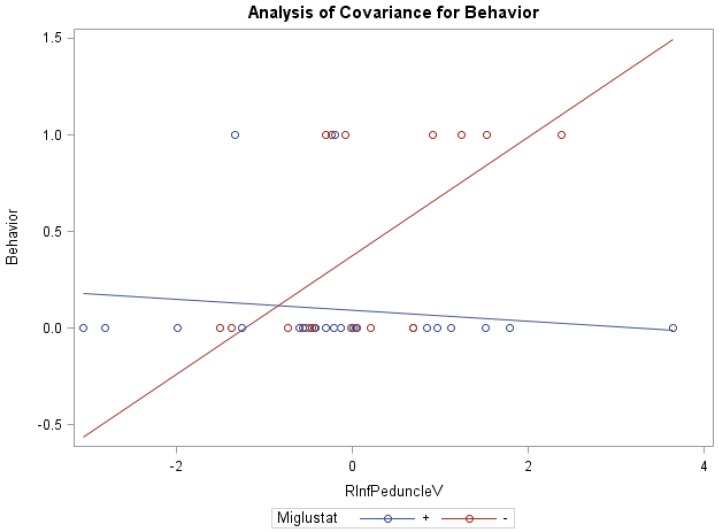
ANCOVA of behavior, miglustat, and volume of r inferior peduncle.

ANCOVA models of NNSS and miglustat therapy, with the volume of the right/left peduncles as the covariate, were statistically significant (Table 6). Groups receiving versus groups not receiving miglustat therapy were found to have significantly lower swallowing subscores (0.78 vs. 1.95) with the volume of the right inferior peduncle as covariate (*f* = 4.02, *p* < 0.05, R^2^ = 0.26), as well as significantly lower behavior subscores (0.09 vs. 0.39), with the covariate volume of the left white matter (*f* = 4.25, *p* < 0.05, R^2^ = 0.27). Statistically significant interaction effects were noted between miglustat therapy with the volume of the right (Figure 2)/left inferior and right/left middle peduncles for behavior subscores, and with the volume of the right/left white matter and right/left/whole cerebellum for auditory brain response subscores (Supplementary Table S3).

### 3.4. Cerebellar Mean Diffusivity (MD)

The cerebellar MD of the right middle peduncle, right white matter, and right/left/whole cerebellum were positively correlated with NNSS (Table 4). MD of other cerebellar regions were also positively correlated with eye movement, ambulation, speech, swallowing, fine motor skills, memory, and auditory brain stem response (Supplementary Table S1). For example, MD of the right/left middle peduncles, right/left white matter, and right/left/whole cerebellum were positively correlated with speech.

Multiple regression models of NNSS with the MD of the right/left middle peduncles, right white matter, and right/left/whole cerebellum, and age at scan covariate were statistically significant (Table 5). Models of eye movement, ambulation, speech, swallowing, memory, and auditory brain response, with MD of cerebellar regions were also found to be statistically significant. Wilcoxon-Mann-Whitney test results indicated no statistically significant differences in cerebellar MD between those on versus those off miglustat therapy.

**Figure 3:**
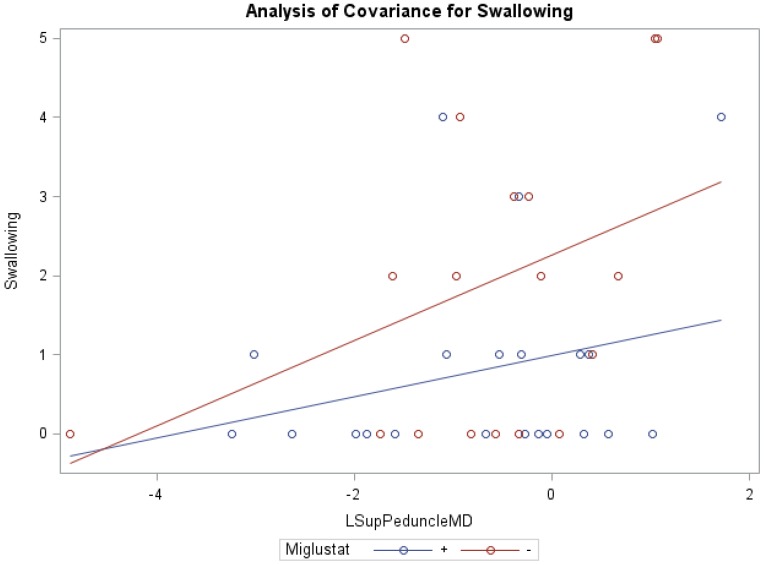
ANCOVA of swallowing, miglustat therapy, and MD of left superior peduncle.

ANCOVA models of NNSS and miglustat therapy, with the covariate MD of the right/left middle peduncles, right white matter, and right cerebellum were statistically significant (Table 6). Similar to cerebellar FA and Volume, ANCOVA indicated that groups receiving versus groups not receiving miglustat therapy differed significantly in their swallowing subscores, with MD of the left inferior (0.83 vs. 1.86; *f* = 3.35, *p* < 0.05, R^2^ = 0.22) and left superior (0.81 vs. 1.88; *f* = 3.17, *p* < 0.05, R^2^ = 0.21; Figure 3) peduncles as respective model covariates. Comparably, with the covariates MD of the right inferior and right/left middle peduncles, right white matter, and right/left/whole cerebellum, the independent effect of miglustat therapy was statistically significant for swallowing subscores (Supplementary Table S3). For eye movement, ambulation, and speech; the independent effect of various cerebellar MD, after accounting for miglustat therapy, were statistically significant.

## 4. Discussion

This is the first study that aims to examine the relationship between cerebellar DTI measurements and NPC1 disease severity. FA and MD are DTI measures of brain microstructural abnormality that are not identified by routine MRI scans. Lower FA and higher MD represent microstructural disorganization in the region examined. Therefore, we hypothesized that decreased FA and increased MD in the cerebellum would be correlated with elevated NIH NPC severity scores, particularly higher motor severity. In our population of NPC1 subjects, deficient cerebellar FA and elevated MD were associated with higher total NPC neurological severity scores, and motor subscores for eye movement, ambulation, speech, swallowing fine motor skills, and cognition. Of the ten cerebellar segments analyzed, superior cerebellar peduncle volume depletion, bilaterally, most consistently correlated with higher scores in ambulation, speech, swallow and fine motor skills, as well as cognition and overall SS, as compared to the other segments.

Our hypothesis that a relationship exists between microstructural disorganization/volume depletion in the cerebellum and NPC disease severity, particularly with motor impairment, was supported by these findings. The data suggest the value of DTI and volume in assessing the clinical markers of NPC1 severity in a pediatric sample. In a previous study looking at the correlation between disease severity and DTI and volume measures in the corpus callosum, it was found that, of the seventeen components that make up the NNSS, the ambulation and motor subscores correlate strongest with disease severity [8]. Another recent study by Walterfang et al. (2013) shows a reduction in NPC grey and white matter cerebellar volumes measures in ten adult NPC patients, when compared to controls. The volume findings in this study did not correlate with symptom duration or severity, but did correlate with saccadic gain and ataxia measures [9]. Our data appears to support these findings in that we report specific clinical measures within the total severity score, such as ambulation, that are correlated with lower cerebellar volume in specific regions; however, there is greater variability in our findings with respect to regions correlating with clinical findings. This may be due to methodological differences between Walterfang and our study, in both cerebellar parcellation and clinical measures of the disease.

An important consideration regarding these results is the effect of NPC severity on motion and the effect of motion on quality of the MRIs obtained. Though the possibility of bias at the level of registration and segmentation effectiveness exists, with motion degradation as a cause of bias, all subjects were MRI scanned under sedation and all motion degraded images were excluded from the study. Therefore, in principal, motion should not factor in as a confounder with FA and higher severity score. We will add these comments to the manuscript discussion.

The specific pathophysiologic mechanism underlying our findings is unknown. Studies of murine models suggest that various pathways play a role, including decreased vitamin E and calcium-binding proteins that increase susceptibility of purkinje cells to oxidative stress, causing decreased dendritic branching and cell death, as well as cellular disorganization on a microstructural level [22,23,24,25]. In these murine models, reactive metabolic changes that lead to alterations in synaptic strength and long-term depression are observed, which are thought to result in progressive purkinje neurotoxicity and initiation of apoptosis and cell death [22,23,26,27].

Our findings support the idea that DTI is a tool for measuring neurodevelopmental impairment. The derived FA and volume may be applied to evaluate NPC disease severity. Previous studies support DTI and volumetric analysis of both grey and white matter as useful modalities for indexing illness stage and monitoring response to treatment [9,12]. DTI analysis of the corpus callosum showed a correlation between volume deficits and increased SS and MSS [8]. Future studies investigating the effects of therapy on NPC may consider using brain DTI data to compare groups. The emerging tool of magnetization transfer ratio (MTR) imaging may also be useful in assessing microstructural brain changes related to clinical deficit [28].

These findings suggest that imaging markers correlate with aspects of disease severity, which contributes to our understanding of the pathophysiology of NPC. However, we are unable to provide enough support for prognostication of disease severity based on our study alone, because NPC’s rare disease status limits the number of subjects required to show correlations between measurements, and there is a significant amount of variance inherent in DTI measures.

Furthermore, our data show that in the inferior peduncle, miglustat has a paradoxical effect at high FA values, as severity scores of patients on miglustat surpass those who are off of the drug. In almost all other regions, as displayed in Figure 3 for swallowing subscore in the superior peduncle, we see miglustat associated with lower severity scores across the spread of FA, MD or volume, except at the very lowest MD levels, closest to where we would expect healthy control subjects to fall. The inferior cerebellar peduncle is composed mostly of the restiform body, a collection of afferent fibers from the posterior spinocerebellar tract of the spinal cord and various tracts from the medulla. The remaining juxtarestiform body is primarily composed of efferent fibers from the vestibulocerebellum to the vestigial nuclei, and from the fastigial nucleus to the uncinate fasciculus [29]. The “equilibrating effect” of miglustat that our data show in the region of the inferior peduncle suggests that the mechanism of action of miglustat may differ in the inferior peduncle compared to the rest of the cerebellum. This may be due to the mixed afferent/efferent cellular make-up of the peduncle, but further research into the mechanism of action of miglustat is warranted to explore this phenomenon further.

Finally, a previous publication investigating effects of volume and FA reduction and increased MD in the corpus callosum on NNSS showed that ambulation, speech, swallow, motor, cognition, memory, seizures and incontinence had a strong negative correlation with whole corpus callosum FA and volume. Our findings in the cerebellum differ in that increased subscores were limited mostly to motor function, with eye movement, ambulation, speech, and swallowing most significantly affected, which would be expected with the known motor function of the cerebellum in the CNS. Similar to the trends observed in the corpus callosum study, these increased NNSS were more often associated with decreased FA and volume, as compared to increased MD. These correlations in the cerebellum are less strong than those found in the corpus callosum study, suggesting that the corpus callosum is more directly affected by the neuronal changes seen with NPC. Alternatively, a broader array of functional impairment is anticipated with corpus callosum impairment, when compared to the cerebellum.

### Study Limitations

One limitation of this study is that our control group for z-score calculation did not have the same scan parameters as our study population, thus direct comparison analysis was not possible. Likewise, no longitudinal study of healthy control subjects was available to provide information about the effects of age on rate of change in FA, volume and MD in the developing child’s brain. Studies have shown that FA changes with age, but it is still unclear if changes vary in different regions at different stages of child development. That being said, two statistical analyses (spearman correlation and multiple regression analysis) were performed to account for age as a confounder. Findings from both analyses support the conclusions of the study. Multiple regression analysis accounted for the covariate of age at scan (or noise) in examining the associations between FA, volume, and MD of various cerebellar regions and NNSS; essentially stronger statistical evidence of an association. Alternatively, ANCOVA highlighted the independent effects of various FA, volume, and MD of cerebellar regions or miglustat therapy, and an interaction effect between cerebellar FA, volume, and MD and miglustat therapy for particular NPC subscores.

The NNSS does not capture all of the motor impairment in NPC patients, and is therefore not a comprehensive evaluation of motor dysfunction. Likewise, the cerebellum does not account for all of the body’s motor function. In our study, these inherently incomplete measures of motor severity, juxtaposed with imaging parameters of one CNS region, limits generalization of findings. Additionally, we relied on an automated parcellation to calculate volume, as a previous study validated this automated method using manual tracing as the gold standard [8].

## 5. Conclusions

In a population of pediatric and adolescent NPC1 patients, impaired cerebellar volume and DTI measures are associated with disease severity, particularly motor dysfunction. Furthermore, our data suggest that the mechanism of action of miglustat may differ in the inferior peduncle compared with the rest of the cerebellum. Longitudinal studies using DTI and neurodevelopmental data should be performed to further advance our scientific understanding of NPC.

## Figures and Tables

**Table 1:** Acquisition Parameters for T2WI and DTI.

Acquisition Parameter	T2WI	DTI
Repetition Time (TR)	5400 ms	6401 ms
Echo Time (TE)	100 ms	60 ms
Field of View (FOV)	220 × 165 mm	224 × 224 mm
Acquisition Matrix	384 × 227	112 × 112
Reconstruction Matrix	512 × 512	128 × 128
In-Plane Resolution	0.43 × 0.43 mm/pixel	1.75 × 1.75 mm/pixel
Acquisition Duration	2:25	2:27
Slice Thickness	5 mm	2 mm
Number of Axial Slices	28	70

**Table 2:** Changes in Patient MPRGE Parameters with Scanner Upgrade (2010).

Scan Parameters	Axial MPRGE < 2010	Axial MPRGE > 2010 (Upgrade)
Repetition Time (TR)	8.2 ms	11.4 ms
Echo Time (TE)	3.8 ms	6.5 ms
FA	8°	6°
Field of View (FOV)	256 mm	220 mm
Acquisition Matrix	256 × 256	256 × 131
Reconstruction Matrix	256 × 256	256 × 256
Slice Thickness	1.0 mm	1.0 mm
Number of Excitations	1	2

**Table 3:** Demographic, Severity Score, and Symptom Data for NPC Patients (*n* = 39).

Subject ^#^	Gender	Age (years)	Total NNSS	Presenting Neurologic Symptom	Age at First Neurologic Symptom (years)	Duration of Neurologic Symptoms (years)	Miglustat Status
1	F	21.2	35	VGSP	9	12.2	−
2	M	7.7	5	Fine Motor Limitation	5	2.7	+
3	F	13.1	33	Clumsy, possible VGSP	2	11.1	+
4	M	5	11	Clumsy, Dysarthria	3	2.0	−
5	M	10	14	Fine Motor Limitation	3	7.0	−
6	M	16.3	18	Dysarthria	3	13.3	−
7	F	11.8	22	Clumsy	1.5	10.3	−
8	F	4	26	Clumsy, VGSP	2	2.0	+
9	M	7.7	7	Abnormal Gait	2	5.7	+
10	M	11.8	18	Fine Motor, Coordination Deficit	6	5.8	+
11	M	5.4	8	Clumsy, Speech Delay	2	3.4	+
12	F	5.2	13	Clumsy	1	4.2	+
13	M	4.7	2	None Reported	N/A	N/A	+
14	M	6.1	7	Clumsy	2	4.1	−
15	F	3.8	3	Clumsy	2	1.7	−
16	F	21.5	35	VGSP	5	16.5	−
17	F	6.7	40	Abnormal gait, fine motor limitation	3	3.7	−
18	F	15.7	1	None Reported	N/A	N/A	+
19	F	6.8	27	Gross motor delay	1.5	5.3	+
20	F	12.6	24	VGSP	5	7.6	−
21	F	16.8	23	Learning difficulty	8	8.8	+
22	M	8.4	46	Clumsy	1.5	6.9	−
23	M	1	1	None Reported	N/A	N/A	+
24	M	1.6	4	None Reported	N/A	N/A	−
25	M	17.2	10	Abnormal Gait	12	5.2	+
26	F	8.1	8	Tremor, fine motor limitation	7	1.1	+
27	M	20.3	14	VGSP, slurred speech	13	7.3	−
28	M	6.6	13	VGSP	3.5	3.1	−
29	M	4	2	None Reported	N/A	N/A	−
30	F	12.7	17	Learning disability	8	4.7	+
31	F	21	18	School problems	5	16.0	−
32	F	15.6	12	Gross motor delay	1.2	14.4	+
33	M	17.2	19	Learning disability	6	11.2	−
34	F	21.9	18	Learning disability	11	10.9	−
35	F	17.3	15	Clumsy	7	10.2	+
36	F	13.3	13	Learning disability	7	6.3	+
37	M	15.2	33	Seizures	6	9.2	+
38	F	11.8	16	VGSP	3	8.7	+
39	M	3.8	2	Gross motor delay	2	1.7	+

F = Female; M = Male; SS = National Institutes of Health, Niemann-Pick disease type C neurological severity score; VGP = Vertical Gaze Palsy; VGSP = Vertical Gaze Supranuclear Palsy; N/A = No data available: No neurologic symptom reported. “+”= on Miglustat and “−” = off Miglustat.

**Table 4:** Spearman Correlations of Cerebellar Measures with NIH NPC Severity Score (NNSS).

Cerebellar Measure	FA		Volume		MD	
	r	p	r	p	r	p
R inferior peduncle	−0.38	0.02	−0.35	0.03		
L inferior peduncle	−0.35	0.03				
R superior peduncle	−0.35	0.03	−0.46	0.003		
L superior peduncle	−0.41	0.01	−0.49	0.001		
R middle peduncle					0.35	0.03
R white matter	−0.33	0.04			0.33	0.04
R cerebellum	−0.42	0.01			0.37	0.02
L cerebellum	−0.35	0.03			0.35	0.03
Whole cerebellum	−0.40	0.01			0.37	0.02

R = Right; L = Left; FA = Fractional Anisotropy; MD = Mean Diffusivity.

**Table 5:** Multiple Regression of NNSS on Cerebellar Measures and Age at Scan.

	Parameter	*t*-Value	*p*<	Model *f*	Model *p*<	R^2^
**FA**						
R inferior peduncle	−78.01	−2.14	0.05	6.01	0.01	0.25
Age at scan	1.16	3.47	0.01			
R white matter	−202.26	−2.48	0.05	6.94	0.01	0.28
Age at scan	1.16	3.67	0.001			
L white matter	−214.00	−2.62	0.05	7.38	0.01	0.29
Age at scan	1.16	3.76	0.001			
**Volume**						
R inferior peduncle	−0.03	−2.48	0.05	6.95	0.01	0.28
Age at scan	1.02	3.52	0.01			
L inferior peduncle	−0.03	−2.32	0.05	6.48	0.01	0.26
Age at scan	0.94	3.33	0.01			
R superior peduncle	−0.03	−3.12	0.01	9.08	0.001	0.34
Age at scan	0.88	3.38	0.01			
L superior peduncle	−0.03	−3.07	0.01	8.87	0.001	0.33
Age at scan	0.85	3.27	0.01			
R middle peduncle	−0.01	−2.29	0.05	6.42	0.01	0.26
Age at scan	1.07	3.50	0.01			
L middle peduncle	−0.003	−2.42	0.05	6.78	0.01	0.27
Age at scan	1.05	3.54	0.01			
R white matter	−0.003	−2.66	0.05	7.50	0.01	0.29
Age at scan	0.94	3.43	0.01			
L white matter	−0.003	−3.21	0.01	9.42	0.001	0.34
Age at scan	1.01	3.78	0.01			
**MD**						
R middle peduncle	16112	2.25	0.05	6.31	0.01	0.26
Age at scan	0.93	3.29	0.01			
L middle peduncle	16918	2.40	0.05	6.71	0.01	0.27
Age at scan	0.95	3.37	0.01			
R white matter	12378	2.18	0.05	6.12	0.01	0.25
Age at scan	0.89	3.20	0.01			
R cerebellum	5248.47	2.10	0.05	5.92	0.01	0.25
Age at scan	0.58	2.03	0.05			
L cerebellum	5035.14	2.08	0.05	5.86	0.01	0.25
Age at scan	0.60	2.12	0.05			
Whole cerebellum	5182.60	2.10	0.05	5.92	0.01	0.25
Age at scan	0.58	2.07	0.05			

R = Right; L = Left; FA = Fractional Anisotropy; MD = Mean Diffusivity.

**Table 6:** ANCOVA of NNSS and Subcores, Miglustat and Cerebellar Measures.

	Type I NNSS	*f*-value	*p*<	Type III NNSS	*f* -value	*p*<	Model *f* value	Model *p*<	Model R^2^
**FA**									
Miglustat	266.74	2.58	0.12	197.29	1.91	0.18	4.63	0.01	0.28
R inferior peduncle	600.57	5.82	0.05	503.01	4.87	0.05			
Age at scan	565.80	5.48	0.05						
Miglustat	266.74	2.59	0.12	263.45	2.56	0.12	4.66	0.01	0.29
L inferior peduncle	530.46	5.15	0.05	442.57	4.30	0.05			
Age at scan	642.40	6.24	0.05						
Miglustat	266.74	2.37	0.14	122.76	1.09	0.31	3.24	0.05	0.22
L superior peduncle	700.03	6.21	0.05	724.33	6.42	0.05			
Age at scan	130.67	1.16	0.29						
**Volume**									
Miglustat	266.74	2.73	0.11	165.04	1.69	0.21	5.57	0.01	0.32
R superior peduncle	1299.66	13.32	0.001	1363.60	13.98	0.00			
Age at scan	63.98	0.66	0.43			1			
Miglustat	266.74	2.48	0.13	85.78	0.80	0.38	3.97	0.05	0.25
L superior peduncle	1011.34	9.40	0.01	846.23	7.87	0.01			
Age at scan	2.12	0.02	0.89						
**MD**									
Miglustat	266.74	2.51	0.13	463.12	4.35	0.05	4.14	0.05	0.26
R middle peduncle	719.61	6.76	0.05	1001.38	9.41	0.01			
Age at scan	334.83	3.15	0.09						
Miglustat	266.74	2.44	0.13	345.29	3.16	0.09	3.73	0.05	0.24
L middle peduncle	715.91	6.55	0.05	920.75	8.43	0.01			
Age at scan	238.39	2.18	0.15						
Miglustat	266.74	2.33	0.14	238.23	2.09	0.16	3.05	0.05	0.21
R white matter	608.32	5.32	0.05	729.96	6.39	0.05			
Age at scan	171.50	1.50	0.23						
Miglustat	266.74	2.31	0.14	175.21	1.52	0.23	2.90	0.05	0.20
R cerebellum	711.98	6.17	0.05	718.60	6.23	0.05			
Age at scan	26.07	0.23	0.64						

R = Right; L = Left; FA = Fractional Anisotropy; MD = Mean Diffusivity.

## Supplementary Materials

The supplementary materials are available online at www.mdpi.com/2079-9721/4/3/29/s1.

## Abbreviations

The following abbreviations are used in this manuscript:

NPC: Niemann-Pick Disease, type C
NPC1: Niemann-Pick Disease, type C1
NPC2: Niemann-Pick Disease, type C2
DTI: diffusion tensor imaging
FA: fractional anisotropy
MD: mean diffusivity
NNSS: NIH NPC severity score
MRI: magnetic resonance imaging
MPRAGE: spatially matched axial magnetization-prepared rapid-gradient echo
T2WI: T2-weighted imaging
LDDMM: Large deformation diffeomorphic metric mapping
ANCOVA: Analysis of covariance

## References

[1] Kemp W.L., Burns D.K., Travis G., Brown T.G. (2008). Chapter 6: Genetic Disorders—Lysosomal Storage Disorders. Pathology: The Big Picture.

[2] Vanier M.T., Latour P. (2015). Laboratory diagnosis of Niemann-Pick disease type C: The filipin staining test. Methods Cell Biol..

[3] Wassif C.A., Cross J.L., Iben J., Sanchez-Pulido L., Cougnoux A., Platt F.M., Ory D.S., Ponting C.P., Bailey-Wilson J.E., Biesecker L.G. (2015). High incidence of unrecognized visceral/neurological late-onset Niemann-Pick disease, type C1, predicted by analysis of massively parallel sequencing data sets. Genet. Med..

[4] Patterson M.C. Overview of Niemann-Pick Disease. http://www.uptodate.com/contents/overview-of-niemann-pick-disease.

[5] Zimran A., Elstein D. (2010). Lipid Storage Diseases. Williams Hematology.

[6] Cluzeau C.V., Watkins-Chow D.E., Fu R., Borate B., Yanjanin N., Dail M.K., Davidson C.D., Walkley S.U., Ory D.S., Wassif C.A. (2012). Microarray expression analysis and identificantion of serum biomarkers for Niemann-Pick disease type C1. Hum. Mol. Gen..

[7] Porter F.D., Scherrer D.E., Lanier M.H., Langmade S.J., Molugu V., Gale S.E., Olzeski D., Sidhu R., Dietzen D.J., Fu R. (2010). Cholesterol oxidation products are sensitive and specific blood-based biomarkers for Niemann-Pick C1 disease. Sci. Transl. Med..

[8] Lee R., Apkarian K., Jung E.S., Yanjanin N., Yoshida S., Mori S., Park J., Gropman A., Baker E.H., Porter F.D. (2014). Corpus Callosum Diffusion Tensor Imaging and Volume Measures are Associated with Disease Severity in Pediatric Niemann-Pick Disease Type C1. Pediatr. Neurol..

[9] Walterfang M., Abel L.A., Desmond P., Fahey M.C., Bowman E.A., Velakoulis D. (2013). Cerebellar volume correlates with saccadic gain and ataxia in adult Niemann-Pick type C. Mol. Gen. Metab..

[10] Walterfang M., Fahey M., Desmond P., Wood A., Seal M.L., Steward C., Adamson C., Kokkinos C., Fietz M., Velakoulis D. (2010). White and gray matter alterations in adults with Niemann-Pick disease type CA cross-sectional study. Neurology.

[11] Walterfang M., Fahey M., Abel L., Fietz M., Wood A., Bowman E., Reutens D., Velakoulis D. (2011). Size and shape of the corpus callosum in adult Niemann-Pick type C reflects state and trait illness variables. Am. J. Neuroradiol..

[12] Trouard T.P., Heidenreich R.A., Seeger J.F., Erickson R.P. (2005). Diffusion tensor imaging in Niemann-Pick Type C disease. Pediatr. Neurol..

[13] Landman B.A., Farrell J.A., Jones C.K., Smith S.A., Prince J.L., Mori S. (2007). Effects of diffusion weighting schemes on the reproducibility of DTI-derived fractional anisotropy, mean diffusivity, and principal eigenvector measurements at 1.5T. Neuroimaging.

[14] Woods R.P., Grafton S.T., Holmes C.J., Cherry S.R., Mazziotta J.C. (1998). Automated image registration: I. General methods and intrasubject, intramodality validation. J. Comput. Assist. Tomogr..

[15] Huang H., Ceritoglu C., Li X., Qiu A., Miller M.I., van Zijl P.C., Mori S. (2008). Correction of B0 susceptibility induced distortion in diffusion-weighted images using large deformation diffeomorphic metric mapping. Magn. Reson. Imaging.

[16] Miller M.I., Beg M.F., Ceritoglu C., Stark C. (2005). Increasing the power of functional maps of the medial temporal lobe by using large deformation diffeomorphic metric mapping. Proc. Natl. Acad. Sci. USA.

[17] Beg M.F., Miller M.I., Trouvé A., Younes L. (2005). Computing large deformation metric mappings via geodesic flows of diffeomorphisms. Int. J. Comput. Vis..

[18] Oishi K., Faria A., Jiang H., Li X., Akhter K., Zhang J., Hsu J.T., Miller M.I., van Zijl P.C., Albert M. (2009). Atlas-based whole brain white matter analysis using large deformation diffeomorphic metric mapping: Application to normal elderly and Alzheimer’s disease participants. Neuroimage.

[19] Faria A.V., Zhang J., Oishi K., Li X., Jiang H., Akhter K., Hermoye L., Lee S.K., Hoon A., Stashinko E. (2010). Atlas-based analysis of neurodevelopment from infancy to adulthood using diffusion tensor imaging and applications for automated abnormality detection. Neuroimage.

[20] Faria A.V., Hoon A., Stashinko E., Li X., Jiang H., Mashayekh A., Akhter K., Hsu J., Oishi K., Zhang J. (2011). Quantitative analysis of brain pathology based on MRI and brain atlases—Applications for cerebral palsy. Neuroimage.

[21] Yanjanin N.M., Vélez J.I., Gropman A., King K., Bianconi S.E., Conley S.K., Brewer C.C., Solomon B., Pavan W.J., Arcos-Burgos M. (2010). Linear clinical progression, independent of age of onset, in Niemann-Pick disease. Am. J. Med. Genet..

[22] Kennedy B.E., LeBlanc V.G., Mailman T.M., Fice D., Burton I., Karakach T.K., Karten B. (2013). Pre-symptomatic activation of antioxidant responses and alterations inglucose and pyruvate metabolism in Niemann-Pick Type C1-deficient murine brain. PloS ONE.

[23] Klein A., Maldonado C., Vargas L.M., Gonzalez M., Robledo F., de Arce K.P., Muñoz F.J., Hetz C., Alvarez A.R., Zanlungo S. (2011). Oxidative stress activates the c-Abl/p73 proapoptotic pathway in Niemann-Pick type C neurons. Neurobiol. Dis..

[24] Ulatowski L., Parker R., Warrier G., Sultana R., Butterfield D.A., Manor D. (2014). Vitamin E is essential for Purkinje neuron integrity. Neuroscience.

[25] Byun K., Kim D., Bayarsaikhan E., Oh J., Kim J., Kwak G., Jeong G.B., Jo S.M., Lee B. (2013). Changes of calcium binding proteins, c-Fos, and COX in hippocampal formation and cerebellum of Niemann-Pick type C mouse. J. Chem. Neuroanat..

[26] Sun C.L., Su L.D., Li Q., Wang X.X., Shen Y. (2011). Cerebellar long-term depression is deficient in Niemann-Pick type C disease mice. Cerebellum.

[27] Elrick M.J., Pacheco C.D., Yu T., Dadgar N., Shakkottai V.G., Ware C., Paulson H.L., Lieberman A.P. (2010). Conditional Niemann-Pick C mice demonstrate cell autonomous Purkinje cell neurodegeneration. Hum. Mol. Gen..

[28] Zaaraoui W., Crespy L., Rico A., Faivre A., Soulier E., Confort-Gouny S., Cozzone P.J., Pelletier J., Ranjeva J.P., Kaphan E. (2011). In vivo quantification of brain injury in adult Niemann-Pick Disease Type C. Mol. Gen. Metab..

[29] Mancall E. (2011). Cerebellum. Gray’s Clinical Neuroanatomy: The Anatomic Basis for Clinical Neuroscience.

